# Effectiveness of intra-thecal methotrexate in refractory Anti-N-methyl-d-aspartate receptor encephalitis

**DOI:** 10.1186/s12883-023-03301-8

**Published:** 2023-07-07

**Authors:** Raid Hommady, Abdullah Alsohibani, Ruba Alayed, Abdulaziz Alshehri, Ahlam AbuMelha, Lama Aljomah, Khalid Hundallah, Mohammed Almuqbil, Waleed Altuwaijri, Ahmad Alrumayyan, Muhammad Talal Alrifai, Duaa Mohammed Baarmah

**Affiliations:** 1 College of Medicine, King Saud bin Abdulaziz University for Health Sciences (KSAU-HS), Riyadh, Saudi Arabia; 2 Division of Pediatric Neurology, Department of Pediatrics, King Abdullah Specialist Children’s Hospital (KASCH), National Guard Health Affairs (NGHA), Riyadh, Saudi Arabia; 3 Ministry of National Guard, King Abdullah International Medical Research Center (KAIMRC), Riyadh, Saudi Arabia; 4 Department of Pediatric Neurology, Prince Sultan Military Medical City, Riyadh, Saudi Arabia; 5 Department of Pediatrics, King Abdullah Bin Abdulaziz University Hospital, Princess Nourah bint Abdulrahman University, Riyadh, Saudi Arabia

**Keywords:** Refractory NMDA encephalitis1, Autoimmune encephalitis2, Methotrexate3, Intra-thecal immunotherapy4

## Abstract

**Background:**

Anti-N-methyl-d-aspartate “anti-NMDA” receptor encephalitis is one of the most common autoimmune encephalitis for which first- and second-line therapies have been recommended following international consensus. However, some refractory cases do not respond to the first- and second-line therapy and require further immune-modulatory therapies such as intra-thecal methotrexate. In this study, we reviewed six confirmed cases of refractory anti-NMDA receptor encephalitis from two tertiary centers in Saudi Arabia that required escalation of treatment and received a six-month course of intra-thecal methotrexate. The aim of this study was to evaluate the effectiveness of intra-thecal methotrexate as immunomodulatory therapy for refractory anti-NMDA receptor encephalitis.

**Methods:**

We retrospectively evaluated six confirmed cases of refractory anti-NMDA receptor encephalitis who did not improve after first- and second-line therapy and received monthly intra-thecal methotrexate treatment course for six consecutive months. We reviewed patient demography, underlying etiologies, and compared their modified Rankin score prior to receiving intra-thecal methotrexate and six months after completing the treatment.

**Results:**

Three of the six patients showed a marked response to intra-thecal methotrexate with a modified Rankin scale of 0–1 at 6-month follow-up. None of the patients experienced any side effects during or after intra-thecal methotrexate treatment, and no flareups were observed.

**Conclusion:**

Intra-thecal methotrexate may be a potentially effective and relatively safe escalation option for immunomodulatory therapy of refractory anti-NMDA receptor encephalitis. Future studies on intra-thecal methotrexate -specific treatment regimens may further support its utility, efficacy, and safety in treating refractory anti-NMDA receptor encephalitis.

## Introduction

Autoimmune encephalitis is a group of immune-mediated inflammatory disorders of the brain, often involving cortical or deep gray matter with or without involvement of the white matter, meninges, or the spinal cord [1]. Autoimmune encephalitis is clinically challenging due to multiple factors such as overlap in the clinical presentation, neuroimaging, and laboratory findings of many forms of autoimmune and infectious encephalitis [2].

Anti-N-Methyl-D-Aspartic Acid (anti-NMDA) encephalitis is the most frequent and best-defined autoimmune encephalitis, considered potentially fatal [3, 4]. It was first diagnosed in 2007 [5]. The pathophysiology of anti-NMDA autoimmune encephalitis is characterized by the presence of immunoglobulin G “IgG” autoantibodies against the GluN1 subunit of the NMDA receptor in the CNS [6, 7].

Autoimmune encephalitis can be complicated and difficult to diagnose initially since it can be associated with many syndromes and different underlying etiologies [8]. However, these patients usually present with either acute, subacute, or chronic fluctuations in the level of consciousness with memory changes and affected cognition that might eventually end up in coma [8].

Clinical presentation of patients with anti-NMDA autoimmune encephalitis can resemble other autoimmune encephalitis with clinical presentations ranging from mild-moderate to severe, with mortality between 5% and 11% [3, 9] Anti-NMDA autoimmune encephalitis is usually characterized by the presence of oro-facial dyskinesia and acute or subacute neuropsychiatric and behavioral manifestations [10].

International consensus recommendations for the treatment of pediatric NMDAR antibody encephalitis have been established to treat patients with autoimmune encephalitis [11]. This management protocol starts with first-line therapy, which involves intravenous administration of methylprednisolone with a tapering plan of prednisolone [1, 11]. Additional first line therapy is therapeutic plasma exchange and/or intravenous immunoglobulin (“IVIG”). Second line therapy consists of Rituximab or Cyclophosphamide. The escalation of management includes Tocilizumab [11, 12].

However, some of the patients diagnosed with severe anti-NMDA autoimmune encephalitis may have slight improvement or no improvement even after reaching the escalation drug and maintenance medications [13, 14].

Intrathecal Methotrexate was considered in multiple studies that did not report any significant side effects or adverse events after a 1year follow-up, and it has been used on very severe cases to prevent further clinical deterioration with reported response [14–17].

To date, no clear evidence on the use of intrathecal Methotrexate on patients with severe anti-NMDA autoimmune encephalitis exists.

This study aimed to retrospectively report our experience with pediatric refractory anti-NMDA autoimmune encephalitis who failed the evidence-based treatment choices and received intrathecal Methotrexate and demonstrate their clinical response.

## Patients and methods

This study was conducted in two well-known tertiary centers in Saudi Arabia: King Abdullah Specialized Children’s Hospital (KASCH), Riyadh, KSA and Prince Sultan Military Medical City, Riyadh, KSA. We included all patients diagnosed with severe and refractory anti-NMDA autoimmune encephalitis who did not respond to first- and second-line treatment and received intrathecal Methotrexate. First-line therapy includes a course of IV methylprednisolone with tapering plan of prednisolone followed by Therapeutic Plasma Exchange and/or Intravenous Immunoglobulins “IVIG.” Second-line therapy consisted of Rituximab or Cyclophosphamide. Further escalation of management included administration of Tocilizumab. In this study, we included all pediatric patients below 14 years of age who were clinically diagnosed with refractory anti-NMDA autoimmune encephalitis and showed unsuccessful recovery after first- and second-line immunotherapy and required intrathecal Methotrexate injection in their treatment course.

Upon review of health care centers in the Gulf region, 6 patients were added to our study, who did not improve after first- and second-line immunotherapy treatment and thus needed an escalation therapy; therefore, they received intrathecal Methotrexate. Four of these patients were recruited from King Abdullah Specialized Children’s Hospital at Ministry of National Guard Health Affairs, KSA, and the other two were recruited from Prince Sultan Military Medical City, KSA.

This was a descriptive chart review study in which electronic medical records systems were reviewed; BestCare system, which is the patient database used at King Abdullah Specialized Children’s Hospital “four cases were included,“ and healthcare system in Prince Sultan Military Medical City “two cases were found.“ The patients were diagnosed with refractory anti-NMDA autoimmune encephalitis which required intrathecal methotrexate injection.

### Intrathecal Methotrexate regimen

The study participants received monthly intrathecal Methotrexate for 6 months with dosing of 8 mg for patients between 1 and 2 years of age, 10 mg for patients between 2 and 3 years of age, and 12 mg for patients > 3 years old. All six doses of IT-MTX were followed by 20 mg IV methylprednisolone.

Standard protocol approvals were established, medication registration was approved, and patient electronic consent for methotrexate injection was signed by parents of all patients, including all possible side effects. Procedural consent was also obtained to proceed with lumbar puncture.

## Result

From January 2015 to October 2022, we enrolled six patients with refractory NMDA encephalitis in this study. The median age was 4.5 years (ranging between 20 months to 9 years), with male predominance of 2:1. Three patients “cases 1,2,3” had prodromal symptoms of fever and upper respiratory tract infection, one patient “case 4” had left-sided focal seizures, and two patients “cases 5 and 6” had excessive sleepiness and behavioral changes. The varying degrees of disease progression into encephalopathy ranged from days to weeks. The three patients “cases 1,2,3” with prodromal symptoms of fever and URTI had relatively more rapid progression to encephalopathy within 7 days, 4 days, and 2 days respectively, moreover, one patient “case 6” with initial presentation of behavioral changes progressed to encephalopathy in 6 days, while the remaining two patients “cases 4 and 5” progressed to encephalopathy after approximately three weeks. Table 1. All patients were placed on first-line therapy within the first week of presentation, including IV methylprednisolone followed by plasma exchange and IVIG, with no significant clinical improvement; thus, second-line treatment with Rituximab was attempted. Escalation therapy, such as Tocilizumab, was administered in three of the six patients. Although all six patients received first- and second-line treatment, they had a Modified Rankin Score of 5 and remained encephalopathic, with persistent choreoathetotic movements and oro-facial dyskinesia along with irritability and insomnia with high NMDA titers in CSF. All six patients received an Intrathecal Methotrexate regimen of single intrathecal age-dependent dosing once monthly for six months (Table 2) with repeated NMDA titer post therapy. Three of the six patients “cases 4, 5 and 6” had significant improvement, with “cases 5 and 6” having a mRS of 0 and “case 6” had a mRS of 1 at 6 months follow-up (Table 3) with resolved choreoathetotic movement and orofacial dyskinesia with improved sleeping and irritability; the three patients did not have any underlying viral etiology. All six cases had focal unaware seizures requiring initiation of anti-epileptic medication. Fortunately, five out of the six cases responded to first and second anti-seizure medications and achieved seizure freedom for at least 1 year follow up. Case 2 is the only patient that was still having uncontrolled seizures on appropriate dual anti-epileptic medications and started to have generalized atonic seizures requiring a third anti-seizure medication with some degree improvement in decreasing seizure frequency to once weekly. Cases 1,2 and 3 had very limited or no response to IT-MTX and were found to have an underlying viral trigger. Case 3 was diagnosed with HSV-encephalitis triggered anti-NMDA encephalitis and had slight transient improvement in terms of encephalopathy after IT-MTX. She started to follow objects with her eyes, with observed biochemical improvement and decreased CSF NMDA titer from 1:2 before IT-MTX to 1:1 after the course of therapy; however, the patient had a relapse few weeks after completing the course of IT-MTX in the form of worsening dystonia and irritability. The other two patients “Cases 1 and 2” had an underlying viral etiology of Rhinovirus and Human Herpes simplex both remained static with a mRS of 5 throughout the 6-month duration of the IT-MTX course and at 6 months follow-up post treatment, with no change in NMDA titer. None of the patients had any tumor with normal initial brain MRI. Apart from the patient “case 4” whose initial presentation was focal seizures with EEG showing bitemporal epileptiform discharges, all other five patients showed diffuse slowing on their first EEGs followed by focal epileptiform discharges on follow-up EEGs. None of the six patients reported any immediate or intermediate side effects of methotrexate.

**Table 1:** Clinical Presentation of Six patients with Refractory Anti-NMDA Receptor Autoimmune Encephalitis

Case	Case 1	Case 2	Case 3	Case 4	Case 5	Case 6
Age at onset	2 years	2 years	20 months	4 years	9 years	8 years
Gender	Female	Male	Female	Male	Male	Male
Initial symptoms	Fever	Fever	Fever	Seizure	Behavioral changes	Behavioral changes
Seizures	Focal unaware seizure	Focal unaware seizure	Focal unaware seizure	Focal unaware seizure	Focal unaware seizure	Focal unaware seizure
Seizure control	Yes	No	Yes	Yes	Yes	Yes
Psychiatric symptoms	Yes	Yes	Yes	Yes	Yes	Yes
Movement disorders	Yes	Yes	Yes	Yes	Yes	Yes
Time from onset to encephalopathy	7 days	4 days	2 days	20 days	21 days	6 days
Viral etiology	HHV6	Rhinovirus	HSV1	None	None	None
Initial EEG	Diffuse slowing	Diffuse slowing	Diffuse slowing	Diffuse slowing and bitemporal discharges	Diffuse slowing	Diffuse slowing
MRI brain	*	Normal	**	Mild brain volume loss	Normal	Normal
CSF						
WBC	110	9	41	1	23	14
Protein	Normal	Normal	Normal	Normal	Normal	Normal
Initial NMDA titer	1:640	1:640	1:81	1:128	1:128	1:160
NMDA titer post IT-MTX	1:640	1:640	1:1	Not repeated	1:10	Not done
Antiviral treatment	Yes “Gancyclovir”	No	Yes “Acyclovir”	No	No	No
Duration prior to initiation of IT-MTX	13 months	6 months	42 months	6 months	5 months	8 months
Initial intensive care unit admission	7 days	8 days	7 days	11 days	7 days	73 days
Total length of admission	32 months	15 months	6 years	14 months	2 months	6 months

* High FLAIR signal in the bilateral medial and posterior thalami, bilateral frontal and parietal cortex

** Atrophy and encephalomalacia involving temporal, inferior frontal and limbic system bilaterally, more on the right side.

**Table 2:** Received treatment for Refractory Anti-NMDA-R encephalitis

Data	Case 1	Case 2	Case 3	Case 4	Case 5	Case 6
IV Methylprednisolone	Yes	Yes	Yes	Yes	Yes	Yes
IVIG	Yes	Yes	Yes	Yes	Yes	Yes
Plasma Exchange	Yes	Yes	Yes	Yes	No	No
Rituximab	Yes	Yes	Yes	Yes	Yes	Yes
Cyclophosphamide	No	No	No	No	No	No
Tocilizumab	No	Yes	Yes	Yes	No	No
IT-MTX	Yes	Yes	Yes	Yes	Yes	Yes

**Table 3:** Modified Rankin Score

Data	Case 1	Case 2	Case 3	Case 4	Case 5	Case 6
At presentation	5	5	5	3	3	3
After first line immunotherapy	5	5	5	5	5	5
After second line immunotherapy	5	5	5	5	5	5
Before IT-MTX	5	5	5	5	5	5
4 weeks after IT-MTX	5	5	5	3	3	3
6 Months after completing IT-MTX	5	5	5	1	0	0

## Discussion

Anti-NMDA encephalitis is the most common autoimmune encephalitis [18]. It was first discovered in 2007. Since then, multiple retrospective and prospective studies have been conducted to reach a consensus for its management. Intravenous Methylprednisolone along with Plasma Exchange and IVIG were considered the first-line therapy. This is followed by rituximab and cyclophosphamide as second-line choices [13]; however, cases of autoimmune encephalitis refractory to first- and second-line therapies have been reported [15].

The decision in our group for first-line therapy was made using available evidence. All patients received IV Methylprednisolone and IVIG as first-line therapy. Most of our patients had no significant clinical improvement after first-line therapy, despite that plasma exchange (PLEX) was used in 4 of the 6 patients. The choice of second-line therapy was unanimous for all patients; rituximab was used.

Recent studies have suggested that one of the causes of failure of first- and second-line therapy is the limited blood-brain barrier penetration for immunosuppressive medications, indicating the need for intrathecal immunosuppressive medication [19, 20].

One of the currently tried medications is intrathecal Methotrexate. It is an FDA-approved anti-folate metabolite used in chemotherapy and immunosuppression in autoimmune diseases. In autoimmune diseases, it plays a role in the repression of T-cell activation, downregulation of B-cell, increasing activated CD-95T cells sensitivity, repression of methyltransferase activity, and inhibition of the binding of beta-1 interleukin to its cell surface receptor [20, 21].  A variety of neurologic complications can result from IT-MTX, including aseptic meningitis, delayed leukoencephalopathy, acute encephalopathy, and transverse myelopathy [22, 23]. Another reported complication of Intrathecal Methotrexate is sub-acute neurotoxicity with EEG finding of Delta brush [24]. Symptoms can be treated with dextromethorphan, which is a non-competitive antagonist of N-methyl-D-aspartic acid [25].

The early use of immunotherapies was and is still a true concern with regard to the safety and tolerance of those medications. A limited number of studies have been conducted to assess the use of intrathecal Methotrexate in refractory NMDA encephalitis, although they were limited by the small sample size, lack of reported immediate side effects, and lack of complications at 1 year post IT-MTX follow up [26].

Intrathecal immunotherapy in the literature was considered in children who showed an insufficient response or relapse to first- and second-line immunotherapy and with a mRS score ≥ 3. In our experience, all of our patients had a greater severity with a mRS score of 5 after first-line immunotherapy. All patients had a prolonged ICU stay and required invasive mechanical ventilation. We used intrathecal methotrexate as an escalation therapy, which showed clinical and biochemical responses in some of the patients. We assessed clinical improvement using the Modified Rankin scale, which was reduced to 1 or 0 for cases 4, 5, and 6. The other patients (cases 1, 2, and 3) had poor mRS scores of 5. However, it is worth mentioning that three of these patients (cases 1, 2, and 3) had prior viral encephalitis, and fortunately did not develop a relapse or flare-up during IT MTX treatment or follow-up period [15, 16].

The recommendation about the timing of escalation therapy in NMDA encephalitis is dependent on multiple factors such as severity, age, and treating center’s experience. The challenge continues as well for choosing the medication whether Tocilizumab, Mycophenolate mofetil, Azathioprine, or IT-MTX [27, 28].

There is no established protocol for intrathecal injection, and various dosing regimens have been used in the literature to provide different outcomes. In a prospective study of four patients with refractory NMDA encephalitis who received intrathecal methotrexate injection on a weekly basis for 4 weeks with improvement in Modified Rankin Score of 0 at 12 months follow-up in three enrolled patients who retained their baseline neurological function with no reported side effects or flarups, one patient showed no clinical response and died secondary to neurological complications [15]. In another study suggesting administration of intrathecal Methotrexate on a monthly basis over 6 months, all included patients achieved a good response with a mRS of 1–2 at 6 months follow-up with no reported side effects at follow up [16]. IT-MTX can be used as adjunctive therapy with other immune-modulatory therapies like Rituximab or Mycophenolate with marked clinical improvement in patients with refractory NMDA receptor encephalitis [14, 17].

## Conclusion

This study included six patients diagnosed with refractory anti-N-Methyl-D-Aspartate Receptor Encephalitis who failed to respond to first- and second-line immunotherapy, required escalation of immunomodulatory therapy, and received intrathecal Methotrexate injection every month for 6 consecutive months. Three of the six patients had a marked response to IT-MTX with a modified Rankin Scale of 0–1 at 6-month follow-up. The three remaining patients were found to have underlying viral etiology had poorer response to IT-MTX. Although the number of included patients was small, none of them experienced any side effects during, or after IT-MTX treatment, and there were no flareups. Further large-scale studies to establish the efficacy, tolerability, indications, and treatment regimen of IT-MTX in refractory anti-NMDA encephalitis are warranted.

### Limitations

This study had some limitations. First, some patients did not receive similar baseline treatment, as options of first- and second-line treatment were made as per physician/hospital guidelines and availability. Second, this was a retrospective study; there was lack of some biochemical results. For example, the post-treatment NMDA titer was not done for all enrolled patients. Third, the sample size was small, which diminished the statistical power of the results.

## Data Availability

All data used in this study are available from the corresponding author on reasonable request.
